# Tailored message interventions versus typical messages for increasing participation in colorectal cancer screening among a non-adherent population: A randomized controlled trial

**DOI:** 10.1186/s12889-016-3069-y

**Published:** 2016-05-24

**Authors:** Kei Hirai, Yoshiki Ishikawa, Jun Fukuyoshi, Akio Yonekura, Kazuhiro Harada, Daisuke Shibuya, Seiichiro Yamamoto, Yuri Mizota, Chisato Hamashima, Hiroshi Saito

**Affiliations:** Graduate School of Human Sciences, and Graduate School of Medicine, Osaka University, 2-2, Yamadaoka, Suita-shi, Osaka, 565-0871 Japan; Department of Health and Social Behavior, School of Public Health, The University of Tokyo, Bunkyo-ku, Tokyo Japan; Cancer Scan, Tokyo, Japan; Graduate School of Human Development and Environment, Kobe University, Kobe, Japan; Cancer Detection Center, Miyagi Cancer Society, Miyagi, Japan; Public Health Policy Research Division, Research Center for Cancer Prevention and Screening, National Cancer Center, Tokyo, Japan; Screening Assessment and Management Division, National Cancer Center, Tokyo, Japan

**Keywords:** CRC screening, Tailored intervention, Cancer worry, Cost-effectiveness, Non-adherent population

## Abstract

**Background:**

The purpose of this study was to examine the effectiveness and cost-efficiency of a tailored message intervention compared with a non-tailored message intervention for increasing colorectal cancer (CRC) screening rates among a non-adherent population, in a community-based client reminder program.

**Methods:**

After a baseline survey for psychological segmentation, 2140 eligible individuals were randomly assigned either to a group with a tailored matched-message condition (*N* = 356), a group with a non-tailored unmatched-message condition (*N* = 355), or to two control groups, one using a typical message with a professional design (*N* = 717) and one without a professional design (*N* = 712). The main outcome measure was attendance rates in a community-organized CRC screening program within five months of receiving a print reminder.

**Results:**

There was a significant difference in fecal occult blood test (FOBT) attendance rates at follow-up assessments between the tailored matched-message condition (14.0 %) and the control (9.9 %; OR = 1.48, *p* = 0.026), while there was no significant difference between the unmatched-message condition (11.0 %) and the control (OR = 1.12, *p* = 0.558), and between the matched-message condition and the unmatched-message condition (OR = 1.32, *p* = 0.219). The cost of a one-person increase in FOBT screening was 3,740 JPY for the tailored matched-message condition, while it was 2,747 JPY for the control.

**Conclusions:**

A tailored-message intervention for segmented individuals designed to increase CRC screening rates in a community-based client reminder program was significantly effective compared to a usual reminder, but not more effective than an unmatched message in a randomized controlled trial, and was not sufficiently effective to highlight its value from a cost perspective. Therefore, the tailored intervention including target segmentation needs to be improved for future implementation in a CRC screening program for a non-adherent population.

**Trial registration:**

UMIN Clinical Trials Registry UMIN000004384. Date of Registration: March 2011.

**Electronic supplementary material:**

The online version of this article (doi:10.1186/s12889-016-3069-y) contains supplementary material, which is available to authorized users.

## Background

Decreases in the incidence and mortality rates of colorectal cancer are public health priorities in developed countries. Among the rankings of cancers in Japan, the incidence of colorectal cancer (colon and rectum) is the fourth most common in men (115.9 per 10,000 population) and the second most common in women (80.5 per 100,000 population) [1], and colorectal cancer mortality is the third highest in men (42.9 per 100,000 population) and first highest women (34.6 per 100,000 population) among the mortality rates for various types of cancer [2]. The implementation of an organized and high-quality colorectal cancer (CRC) screening program can be an effective tool to reduce the incidence and mortality rates of the disease, based on evidence from controlled trials [3, 4]. Despite the potential for mortality prevention, the use of CRC screening procedures through the fecal occult blood test (FOBT) remains low, with one study showing only 34 % of the US population to have participated in CRC screening according to recommendations [5, 6], and another showing only 38 % of the Japanese population to have participated in 2013 [7].

Interventions using client reminders, including recall letters, may increase the rate of FOBT [8]. In addition, one effective evidence-based strategy that is often employed to increase cancer screening (e.g., mammography) is the provision of tailored interventions [9, 10]. Tailored interventions include individual assessments and the provision of tailored messages in print, by telephone, or in person. Psychological theories and models of health behavior change have been applied to developing the key messages of tailored materials [8]. Our prior trial developed intervention materials tailored by intentions (a key construct of the Theory of Planned Behavior [11]) and cancer worries [12], and revealed that an intervention using them can increase the rate of participation in mammography [13]. However, tailored interventions require customized materials for individual or in-person counseling, which adds costs. In terms of community-based cancer screening programs, the cost-effectiveness of the intervention is essential. Low-cost client reminders combined with tailored messages for target characteristics are optimal for community-based interventions, and will enable effective use of CRC screening.

The purpose of this randomized controlled study was to examine the effectiveness and cost-efficiency of a tailored message intervention based on a social marketing approach. We compared the tailored message intervention with a non-tailored message intervention (sending unmatched tailored messages) and control message, in terms of the effect on CRC screening rates among a non-adherent population, as part of a community-based client reminder program for CRC screening in a Japanese community sample. The effectiveness and cost-efficiency of the intervention was demonstrated. As far as we know, there are no available Japanese intervention studies to increase participation in CRC screening.

## Methods

### Design

This study used a prospective randomized controlled design in a Japanese community setting.

### Setting

Onomichi City, Hiroshima Prefecture, Japan has been providing mass CRC screening, involving an annual two-day fecal immunological test, for more than 5500 local residents annually, with residents aged 40 years or older being targets. Data from the 2010 CRC screening program were used for secondary analysis in the present study. The population of the city was approximately 148 000 in 2010. Co-payment for the test was 600 JPY (approximately 7 USD) for those aged 40 to 69 years and 200 JPY (approximately 2 USD) for those aged 70 years and older. The FOBT test was provided at 86 clinics designated by the local government authorities throughout this city.

### Participants and procedures

Individuals selected for inclusion in the 2010 CRC screening program in Onomichi city met the following criteria: (a) no recorded participation in FOBT in the previous 12 months as part of screening programs organized and conducted by the local government, (b) an age of 46–66 years, and (c) membership in Japan’s national health insurance program according to the population registry of the city.

**Fig. 1:**
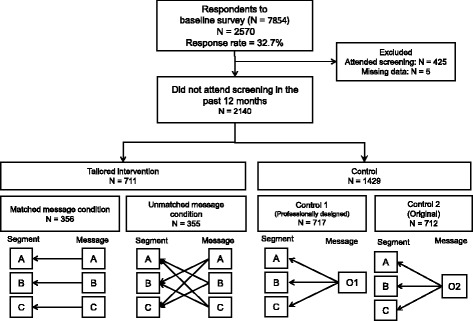
Flow diagram of the trial process

The flow of participants is described in Fig. 1. In October 2010, 7854 individuals were identified. A baseline mail survey was conducted to obtain information on FOBT use, psycho-socio-demography, and history of disease (see Measures). The study’s aim and plan were announced on the local government’s Website. Of the 7854 individuals who received the mail survey, 2570 replied (response rate of 32.7 %). Four hundred and thirty individuals were subsequently excluded based on the eligibility criteria. Following the baseline survey, 2140 eligible individuals were randomly assigned to either tailored intervention groups with a matched-message condition (*n* = 356) or an unmatched-message condition (*n* = 355), or to two control groups, one involving a professionally designed typical message (control group 1, *n* = 717) and one with a typical message that was not professionally designed (control group 2, *n* = 712). In November 2010, after random allocation, the city mailed printed reminders with tailored (matched and unmatched) and control (not professionally designed and professionally designed) messages to prompt study participants to participate in FOBT. FOBT was available from November 2010 to March 2011.

### Intervention

The tailored intervention had two components: (1) an individual assessment, and (2) an assessment-based tailored message.

#### Individual assessment

As a first step in tailored interventions, a variety of variables such as age, risk, and barriers to screening, as well as psychological variables based on theoretical models, have been used as part of individual assessments. According to evidence from our previous trial for breast cancer screening, this study employed two theory-based variables: intention to undergo screening and cancer worry. Intention to have an FOBT is a theoretical construct based on the theory of planned behavior [10], whereby one of the strongest immediate determinants of behavior is a person’s intention to perform it. Cancer worry is defined as an “emotional reaction to the threat of cancer” [11]. In our previous tailored intervention trial for breast cancer screening, a tailored print reminder using two theory-based variables was an effective and cost-efficient strategy for improving breast cancer screening rates among non-adherent individuals [12]. Therefore, we proposed that two theory-based variables would be valid for a tailored intervention for FOBT.

Based on these two variables, we identified the following three segments among non-adherent people: those with high intention and low or high worry (Segment A), those with low intention and high cancer worry (Segment B), and those with low intention and low cancer worry (Segment C).

#### Tailored message

Three types of tailored persuasive statements that were suited to each segment were developed through formative research by the study researchers and social marketers. The formative research used in a social marketing approach is the basis for developing effective messages and materials for influencing behavior change [14–16], and helps researchers identify and understand the characteristics, interests, behaviors, and needs of target populations that influence their decisions and actions. Table 1 lists an example of each tailored persuasive statement. For people with high intention (Message A), clear information about where/when/how they could receive screening was conveyed. For individuals with low intention and high cancer worry (Message B), a gain-framed message that emphasized the benefits of receiving FOBT was conveyed. For people with low intention and low cancer worry (Message C), a loss-framed message that emphasized the costs of not receiving a mammogram was conveyed. These gain- and loss-framed messages were developed based on the framing postulate of prospect theory [16], which states that the choice of a risky option, such as cancer screening, may depend on whether the option is positively or negatively framed. Individuals avoid taking risks when considering gains, but prefer taking risks when considering losses [17].

**Table 1:** Tailored persuasive message examples

Message	Group	Type of message sent
A	High screening intention	Clear information about where/when/how they can receive screening
B	Low screening intention/high cancer worry	Gain-framed message:
		“Detecting cancer early can lead to a higher chance of survival”
C	Low screening intention/low cancer worry	Loss-framed message:
		“Not detecting cancer early can increase the risk of fatality”
Control group		Usual reminder:
		“You are due for your cancer screening” “FOBT is needed for colorectal cancer screening”

In addition to the tailored persuasive statements, the print reminders informed individuals that they were due for an FOBT, and included facts about colorectal cancer and FOBT, such as the morbidity and mortality rate of colorectal cancer and the importance of early detection. Three messages were professionally designed.

Three different tailored messages were delivered to each targeted segment, as well as to the other segments (e.g., Message A to Segment A [matched condition]; Message A to Segment B and C [unmatched-message condition]).

#### Typical message for controls

Although no tailored persuasive statements were delivered to the control groups, printed reminders were delivered to inform them that they were due for a FOBT, and general information on screening procedures and colorectal cancer screening was provided (Table 1). The original control message was developed by an officer in the city who had been responsible for CRC screening, based on materials that had been developed by the National Cancer Center in Japan [18]. In order to be equivalent to the professionally designed tailored messages, another control message was developed with a professional design (O1 in Fig. 1). This message contained the same message and contents as the original control message (O2 in Fig. 1). These two control messages were delivered to all three segments in control group 1 and 2.

### Measurements (Additional file 1)

#### FOBT use

The main outcome measure was participation in community-organized colorectal cancer screening within five months of receiving a print reminder. Data on FOBT attendance were collected as part of the standard record-keeping functions of the healthcare facilities designated by the local government. Each facility sent a written notification to the local government after performing an FOBT. This information was then transferred to a medical history form and was used to determine the number of FOBTs undertaken.

In the baseline survey, FOBT use, socio-demographic variables, behavioral intention, family history of CRC, and history of bowel disease were assessed.

#### Psycho-socio-demographic variables

Information on age, sex, education level, subjective economic status, and marital status were obtained. Psychological information, and behavioral intentions derived from the theory of planned behavior [10] for screening and cancer worry [11] were also obtained. Both were measured by a single item: “Do you intend to attend colorectal cancer screening in the next 12 months?” on a 3-point Likert-type scale, and “Do you worry about getting cancer?” on a 4-point Likert-type scale developed in a previous study [19].

### Statistical analysis

Descriptive analyses were performed to summarize the participants’ backgrounds and psychological measurement scores. Firstly, logistic regression analysis was performed to obtain Odds ratio of difference in attendance rates among two control groups. After confirming that there was no significant difference, we pooled one control, and multiple logistic regression analysis with the control serving as the reference group was performed to determine if FOBT uptake differed among the tailored-matched message condition, the unmatched-message condition, and the controls during a follow-up period. We also analyzed the cost-effectiveness of the interventions by dividing the cost by the uptake in the number of FOBT. All analyses were based on intention-to-treat, and were performed using SAS 9.1.3 statistical software by an epidemiologist in authors (YI).

### Ethical considerations

This study was approved by the Institutional Review Board (IRB) of the Miyagi Cancer Society (No.1005) and adopted the principles of the Declaration of Helsinki. The IRB granted an exemption for written informed consent because of the minimal risk associated with the print reminder, and because of a guarantee of at least usual care for all eligible community members by the local government.

## Results

### Baseline characteristics of respondents

Of the 2570 respondents, 425 had attended FOBT in the previous 12 months, and 5 had missing data. Thus, 430 respondents were excluded from the trial. Among the remaining 2140, after excluding those who had attended previous screenings, 425 were in Segment A, 847 were in Segment B, and 868 were in Segment C. Participants were randomly assigned to either the tailored intervention (*n* = 711; matched-message condition: *n* = 356; unmatched-message condition: *n* = 355), Control Group 1 (*n* = 717), or Control Group 2 (*n* = 712). Table 2 presents the baseline demographic data and psychological characteristics of the four study groups. There were no significant differences in any of these variables among the groups (Table 2).

**Table 2:** Baseline demographic and psychological characteristics of the four study groups (*n* = 2140)

		Intervention group				Control group 1 *n* = 717		Control group 2 *n* = 712		*p* value
		Matched message *n* = 356		Unmatched message *n* = 355						
		Mean/ratio	SD	Mean/ratio	SD	Mean/ratio	SD	Mean/ratio	SD	
Intention (scale: 1–3)		2.04	0.59	2.07	0.57	2.03	0.60	2.05	0.59	0.786
Cancer worry (scale: 1–4)		2.52	0.72	2.54	0.75	2.54	0.75	2.53	0.74	0.964
Age	40s	4.8 %	–	4.6 %	–	4.9 %	–	6.1 %	–	0.859
	50s	25.4 %	–	22.3 %	–	24.4 %	–	24.3 %	–	
	60s	69.9 %	–	73.1 %	–	70.7 %	–	69.7 %	–	
Sex	Male	44.2 %	–	39.3 %	–	42.7 %	–	41.5 %	–	0.574
	Female	55.8 %	–	60.7 %	–	57.3 %	–	58.5 %	–	
Marital status	Married	73.9 %	–	70.2 %	–	68.7 %	–	71.8 %	–	0.315
	Unmarried	26.1 %	–	29.8 %	–	31.3 %	–	28.2 %	–	
Education level (scale: 1–5)		2.34	0.99	2.26	0.93	2.33	0.93	2.30	0.91	0.599
Subjective economic status (scale: 1–5)		2.57	0.86	2.66	0.89	2.58	0.89	2.66	0.87	0.258

### Effect of the intervention on FOBT attendance rates

**Fig. 2:**
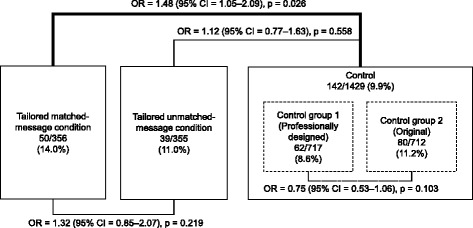
Results for attendance rates and logistic regression for tailored matchedmessage condition, unmatched-message condition, and control

Figure 2 shows the comparison among the matched message condition, the unmatched message condition, and the two control groups (with professional design and without). Firstly, there was no significant difference in FOBT attendance rates in follow-up assessments between Control Group 1 (with professional design) and Control Group 2 (without professional design), and we pooled Control Group 1 and 2 into one control. There was a significant difference in FOBT attendance rates in follow-up assessments between the tailored matched-message condition (14.0 %) and the control (9.9 %; OR = 1.48, *p* = 0.026), while there was no significant difference between the tailored unmatched-message condition (11.0 %) and the control (OR = 1.12, *p* = 0.558), and between the matched-message condition and the unmatched-message condition (OR = 1.32, *p* = 0.219). Table 3 shows attendance rates for the three different segments (A, B, and C) across the intervention conditions. Highest attendance rates were observed for Segment A in the matched-message condition (21.1 %), and the lowest rate was observed for Segment C and the unmatched message condition in the tailored intervention group (6.3 %).

**Table 3:** Differences in FOBT attendance rates among segments for each intervention condition

Segment	Tailored matched-message condition	Tailored unmatched-message condition	Tailored intervention total	Control group 1e	Control group 2	Control total
	*n* = 356	*n* = 355	*n* = 711	*n* = 717	*n* = 712	*n* = 1429
A	21.1 %	15.5 %	18.3 %	13.5 %	14.8 %	14.2 %
B	14.2 %	14.2 %	13.8 %	8.1 %	11.0 %	9.6 %
C	14.0 %	6.3 %	8.4 %	6.8 %	9.7 %	8.3 %
Total	14.0 %	11.0 %	12.5 %	8.6 %	11.2 %	9.9 %

Note: Attendance rate percentages are shown

### Intervention cost and cost-effectiveness

Table 4 shows the specific materials used for the interventions and the costs for each element. While the cost per participant was 524 JPY for the tailored matched-message condition, 523 JPY for the unmatched-message condition, and 273 JPY for both control groups, the cost of one increase in FOBT screening was 3,740 JPY for the tailored matched-message condition, 4,783 JPY for the tailored unmatched-message condition, and 2,747 JPY for the control.

**Table 4:** Cost and cost-effectiveness of the tailored intervention, control group 1, and control group 2 materials

Item		Tailored matched-messagecondition			Tailored unmatched-message condition			Control		
		Unit price (JPY)	Quantity	Total cost (JPY)	Unit price (JPY)	Quantity	Total cost (JPY)	Unit price (JPY)	Quantity	Total cost (JPY)
Individual assessment										
	Questionnaire	30	356	10 680	30	355	10 650			0
	Envelopes	42	356	14 952	42	355	14 910			0
	Postage	175	356	62 300	175	355	62 125			0
	Data entry and analysis	5	356	1780	5	355	1775			0
Overhead costs^a^		10 000	3	30 000	10 000	3	30 000	10 000	12	120 000
Reminder										
	Envelopes	26	356	9256	26	355	9230	26	1,429	37 154
	Printing	43	356	15 308	43	355	15 265	43	1,429	61 447
	Postage	120	356	42 720	120	355	42 600	120	1,429	171 480
Total cost				186 996			186 555			390 081
Cost per participant				524			523			273
Cost per extra CRC				3740			4783			2747

Individual assessment costs in the control were excluded, because individual assessment would not be needed without tailoring procedure

^a^Based on administrative staff salary: 10 000 JPY/day

## Discussion

The key, unique finding of this study was that tailored message interventions aimed at increasing CRC screening rates, as part of community-based client reminder programs, are significantly effective compared to the usual reminder intervention, in a randomized controlled trial. Our intervention was tailored using a social marketing approach, based on respondents’ psychological attributes such as implementation intention and cancer worry, and did not include direct contact such as telephone calls. Although a previous study [20] reported that targeted interventions with tailored mailed message were found to increase colorectal cancer screenings compared to the control without any mailing information, there was no significant difference between standard intervention using mailed messages and tailored intervention. Another study reported that a tailored intervention was not more effective at increasing screening than a public web site or only being surveyed [21].

We could not demonstrate the superiority of a psychologically based tailoring procedure because of no significant difference between the matched-message and the unmatched-message condition (e.g., Messages A & B sent to Segment C). However the study showed that respondents in the tailored-message condition had significantly higher attendance rates of FOBT compared with the control, and that the highest attendance rates were observed for Segment A in the matched-message condition (21.1 %). Furthermore, since there was no significant difference between the professional design and original messages in the control, the design on the reminder might not have an impact on the screening behavior. Therefore, the effectiveness of a tailored-message for each segment based on psychological attributes has been shown and its validity has been partially confirmed.

Although the tailored matched-message condition has significantly larger attendance rates than the control, its effect size has not been large (OR = 1.48). In our previous study regarding breast cancer screening [13], we adopted the same segmentation algorithm, carried out a tailored message intervention, and obtained a significant effect and large size effect size (OR = 4.02) for tailored message interventions; however, the research design of our previous study was not a full factorial (without the unmatched condition and professionally designed control message). There are several possible explanations for the differences in results between our previous study and the current study.

First, the differences may have resulted from differences between CRC screening and breast cancer screening, including demographic characteristics such as targeted age of participants and gender, and general perception of screening and the disease. For example, one study reported that mammographic screening compliance was relatively high (70 %), whereas only 29 % of patients were compliant with FOBT, in a sample of individuals [22]. Similarly, the attendance rate of the tailored-message condition in the previous breast cancer study (19.9 %) was higher than that of the tailored matched message condition in the current study (14.0 %). Therefore, it may be more difficult to increase the uptake of CRC screenings compared to breast cancer screenings without improving segmentation procedures using psychological attributes and demographic characteristics.

Second, the control message (used in the professionally designed version and original version) was developed based on published materials from the Japanese national cancer center. Thus, the content of the message used in the control might have been more powerful for participants than that used in our previous study on breast cancer screening.

The important practical implication of this study was in the cost-effectiveness for the designing community intervention in CRC screening. We obtained the highest attendance rate in Segment A, for which people have a goal and implementation intention (i.e., high readiness for screening and a gain-framed message), similar to our previous mammography study. On the other hand, our study revealed a low attendance rate for Segment C, in which people indicated a low threat of getting cancer, even though they received a loss-framed message. As a meta-analysis showed that gain-framed messages were more likely than loss-framed messages to encourage prevention behaviors [23], using gain-framed messages for general population is reasonable. In addition, the cost-effectiveness of the tailored intervention in this study was not demonstrated. As a study demonstrated that the targeted intervention was more effective and less costly than the tailored intervention [24], the result might not support using tailoring procedures based on psychological segmentation for the intervention in terms of cost-effectiveness. Therefore, sending a reminder with a gain-framed message developed from psychological attributes of certain targeted individuals to all the candidates in the community, without tailoring and segmentation, will contribute to higher attendance rates for CRC screening in a non-adherent population, in a cost-effective way.

This study has several limitations. First, this study was carried out in a suburban city and with people in a limited age range, so the results may not be generalizable to other groups of individuals in different settings. Second, the size of the sample for each intervention group might have been small for detecting detailed differences among the intervention groups. Third, the control message in this study might not represent the message developed by local government officials without a social marketing approach. Fourth, although we calculated the total costs of the interventions, we were not able to consider the cost of building a call-recall system. This might limit the implementation of our method to communities with call-recall systems.

## Conclusions

A tailored-message intervention for segmented individuals designed to increase CRC screening rates in a community-based client reminder program was significantly effective compared to a usual reminder, but not more effective than an unmatched message in a randomized controlled trial, and was not sufficiently effective to highlight its value from a cost perspective. Therefore, the tailored intervention including target segmentation needs to be improved for future implementation in a CRC screening program for a non-adherent population.

### Ethics approval and consent to participate and publication

This study was approved by the Institutional Review Board (IRB) of the Miyagi Cancer Society (No.1005) and adopted the principles of the Declaration of Helsinki. The IRB granted an exemption for written informed consent because of the minimal risk associated with the print reminder, and because of a guarantee of at least usual care for all eligible community members by the local government.

### Availability of data and materials

All data and materials used in this study is publicly available at BMC Public Health’s website as supplementary files (Addtional file 2).

## Additional files

Additional file 1: Questionnaire used in the baseline survey. (PDF 356 kb) Additional file 2: Original data. Data used for analysis. (XLSX 199 kb)

## Abbreviations

CRC: colorectal cancer
FOBT: fecal occult blood test
IRB: Institutional Review Board

## References

[1] Cancer Information Service, National Cancer Center, Japan. Cancer Registry and Statistics. cancer mortality (1958–2014) http://ganjoho.jp/reg_stat/statistics/dl/index.html. Accessed 22 Feb 2016.

[2] Cancer Information Service, National Cancer Center, Japan. Cancer Registry and Statistics. cancer incidence (1975–2011) http://ganjoho.jp/reg_stat/statistics/dl/index.html. Accessed 22 Feb 2016.

[3] Walsh JME, Terdiman JP (2003). Colorectal cancer screening: scientific review. JAMA.

[4] Hewitson P, Glasziou P, Watson E, Towler B, Irwig L (2008). Cochrane systematic review of colorectal cancer screening using the fecal occult blood test (hemoccult): an update. Am J Gastroenterol.

[5] Subramanian S, Klosterman M, Amonkar MM, Hunt TL (2004). Adherence with colorectal cancer screening guidelines: a review. Prev Med.

[6] Vernon SW (1997). Participation in colorectal cancer screening: a review. J Natl Cancer Inst.

[7] Cancer Information Service, National Cancer Center, Japan. Cancer Registry and Statistics.. Cancer screening rate (207–2013). http://ganjoho.jp/reg_stat/statistics/dl/index.html. Accessed 10 Jan 2016.

[8] Baron RC, Rimer BK, Breslow RA (2008). Client-directed interventions to increase community demand for breast, cervical, and colorectal cancer screening: a systematic review. Am J Prev Med.

[9] Albada A, Ausems MGEM, Bensing JM, van Dulmen S (2009). Tailored information about cancer risk and screening: a systematic review. Patient Educ Couns.

[10] Sohl SJ, Moyer A (2007). Tailored interventions to promote mammography screening: a meta-analytic review. Prev Med.

[11] Ajzen I (1991). The theory of planned behavior. Organ Behav Hum Decis Process.

[12] Hay JL, Buckley TR, Ostroff JS (2005). The role of cancer worry in cancer screening: a theoretical and empirical review of the literature. Psychooncology.

[13] Ishikawa Y, Hirai K, Saito H (2012). Cost-effectiveness of a tailored intervention designed to increase breast cancer screening among a non-adherent population: a randomized controlled trial. BMC Public Health.

[14] Maddock JE, Silbanuz A, Reger-Nash B (2008). Formative research to develop a mass media campaign to increase physical activity and nutrition in a multiethnic state. J Health Commun.

[15] Schmidt WP, Wloch C, Biran A, Curtis V, Mangtani P (2009). Formative research on the feasibility of hygiene interventions for influenza control in UK primary schools. BMC Public Health.

[16] Silk KJ, Bigbsy E, Volkman J (2006). Formative research on adolescent and adult perceptions of risk factors for breast cancer. Soc Sci Med.

[17] Tversky A, Kahneman D (1981). The framing of decisions and the psychology of choice. Science.

[18] Cancer Screening Assessment and Management Division RCfCPaS. Colorectal cancer: Guideline for cancer screening. 2010. http://canscreen.ncc.go.jp/guideline/daicyougan.html. Accessed 26 Nov 2012.

[19] Hirai K, Harada K, Seki A (2013). Structural equation modeling for implementation intentions, cancer worry, and stages of mammography adoption. Psychooncology.

[20] Myers RE, Sifri R, Hyslop T (2007). A randomized controlled trial of the impact of targeted and tailored interventions on colorectal cancer screening. Cancer.

[21] Vernon SW, Bartholomew LK, McQueen A (2011). A randomized controlled trial of a tailored interactive computer-delivered intervention to promote colorectal cancer screening: sometimes more is just the same. Ann Behav Med.

[22] O’Donnell S, Goldstein B, Dimatteo MR, Fox SA, John CR, Obrzut JE (2010). Adherence to mammography and colorectal cancer screening in individuals 50–80 years of age: the role of psychological distress. Individuals’s Health Issues.

[23] Gallagher KM, Updegraff JA (2012). Health message framing effects on attitudes, intentions, and behavior: a meta-analytic review. Ann Behav Med.

[24] Lairson DR, DiCarlo M, Myers RE (2008). Cost-effectiveness of targeted and tailored interventions on colorectal cancer screening use. Cancer.

