# Correction to: Tailored message interventions versus typical messages for increasing participation in colorectal cancer screening among a non-adherent population: A randomized controlled trial

**DOI:** 10.1186/s12889-020-09823-x

**Published:** 2020-11-16

**Authors:** Kei Hirai, Yoshiki Ishikawa, Jun Fukuyoshi, Akio Yonekura, Kazuhiro Harada, Daisuke Shibuya, Seiichiro Yamamoto, Yuri Mizota, Chisato Hamashima, Hiroshi Saito

**Affiliations:** 1 Graduate School of Human Sciences, and Graduate School of Medicine, Osaka University, 2–2, Yamadaoka, Suita-shi, Osaka, 565-0871 Japan; 2 Department of Health and Social Behavior, School of Public Health, The University of Tokyo, Bunkyo-ku, Tokyo, Japan; 3 Cancer Scan, Tokyo, Japan; 4 Graduate School of Human Development and Environment, Kobe University, Kobe, Japan; 5 Cancer Detection Center, Miyagi Cancer Society, Miyagi, Japan; 6 Public Health Policy Research Division, Research Center for Cancer Prevention and Screening, National Cancer Center, Tokyo, Japan; 7 Screening Assessment and Management Division, National Cancer Center, Tokyo, Japan

**Correction to: BMC Public Health 16, 431 (2016)**

**https://doi.org/10.1186/s12889-016-3069-y**

It was highlighted that in the original article [1] the Competing interests section was incorrect. This Correction article shows the incorrect and correct Competing interests section.

**Incorrect**

The authors declare that they have no competing interests.

**Correct**

Jun Fukuyoshi and Akio Yonekura are the founders of Cancer Scan Co. Ltd. Yoshiki Ishikawa a director at Cancer Scan Co. Ltd. The other authors declare that they have no competing interests.
