# Supplementary material for: Tailored message interventions versus typical messages for increasing participation in colorectal cancer screening among a non-adherent population: A randomized controlled trial
Source: BMC Public Health. 2016 May 24;16:431. doi: 10.1186/s12889-016-3069-y (PMC4877938; doi:10.1186/s12889-016-3069-y)
Supplement: Additional file 1: — Questionnaire used in the baseline survey. (PDF 356 kb) [file 12889_2016_3069_MOESM1_ESM.pdf]

尾道市大腸がん検診に関する意識調査

記入方法：黒色のペンまたは鉛筆で、◯の中を正確に塗りつぶしてください。

良い例	悪い例
●	✓ ⓘ

問 1 大腸がん検診（便検査）の受診状況についてお聞きします。以下からあなたの状況に最も当てはまるものを1つ選んで◯を塗りつぶしてください。

- ◯ 過去一度も便検査を受けたことがない
- ◯ 過去に受けたことはあるが今年度 (H22.4.1 ～) 受けていない
  最後に受診したのは（　　）年（　　）ヶ月前
- ◯ 今年度 (H22.4.1 ～) に受けた

問 2 今後の大腸がん検診（便検査）の受診に対するお考えについてお聞きします。これから先、便検査を受けるつもりですか？以下から最も当てはまるものを1つ選んで◯を塗りつぶしてください。

- ◯ この 1 年以内に受けるつもりはないし、これから先もずっと受けるつもりはない → 問 3 へ進む
- ◯ この 1 年以内に受けるつもりはないが、いつかは受けたい → 問 3 へ進む
- ◯ この 1 年以内に必ず受けるつもりである → 問 2-1 へ進む

問 2-1 問２で「この１年以内に必ず受けるつもりである」と答えた方にお伺いします。

A. 何月頃に大腸がん検診（便検査）を受けるか決めていますか？

- ◯ 決めている ⟶（　　　　　月頃）
- ◯ 決めていない

B. どこで大腸がん検診（便検査）を受けるか決めていますか？

- ◯ 決めている ⟶（場所　　　　　）
- ◯ 決めていない

→ 問 3 へ進む

問 3 大腸がんになることはどのくらい心配ですか？最も当てはまるものを1つ選んで◯を塗りつぶしてください。

全く心配していない	あまり心配していない	いくらか心配している	かなり心配している
◯	◯	◯	◯

Bk
w00022514_pera 2010年10月21日 8:09:06
Web Printing inc.

最後にあなた自身のことについてお伺いします。

問 4 あなたの性別をお伺いします。

- ◯ 男性
- ◯ 女性

問 5 あなたの年齢をお伺いします。

- ◯ ４０代
- ◯ ５０代
- ◯ ６０代

問 6 あなたが最後に卒業された学校は次のうちどれにあたりますか？

- ◯ 中学校
- ◯ 高等学校
- ◯ 短大・専門学校
- ◯ 大学・大学院
- ◯ その他
- ◯ 答えたくない

問 7 あなたのお宅の暮らしむきは全般的にみて、次のどれにあてはまりますか？

- ◯ かなり苦しい
- ◯ やや苦しい
- ◯ ふつう
- ◯ やや余裕がある
- ◯ 余裕がある
- ◯ 答えたくない

問 8 あなたには現在、配偶者がいますか？

- ◯ いる
- ◯ いない

問 9 あなたの直系のご家族（親や子ども、兄弟姉妹など）で、大腸がんと診断された方はいますか？

- ◯ はい
- ◯ いいえ
- ◯ 答えたくない

問 10 あなたはこれまで、腸や肛門などの病気にかかったことがありますか？

該当する選択肢すべての◯を塗りつぶしてください。（複数回答）

- ◯ なし
- ◯ 大腸ポリープ
- ◯ 痔 (じ)
- ◯ 潰瘍 (かいよう) 性大腸炎
- ◯ 大腸がん
- ◯ 答えたくない

質問は以上です。記入漏れがないかをご確認の上、同封の返信用封筒に入れ、

11 月 13 日（土）までにポストに投函して下さい。

ご協力ありがとうございました。

Bk
w00022513_pera 2010年10月21日 8:08:39
Web Printing inc.
